# Identifying Children With Anxiety Disorders Using Brief Versions of the Spence Children’s Anxiety Scale for Children, Parents, and Teachers

**DOI:** 10.1037/pas0000570

**Published:** 2018-06-14

**Authors:** Tessa Reardon, Susan H. Spence, Jordan Hesse, Alia Shakir, Cathy Creswell

**Affiliations:** 1 School of Psychology and Clinical Language Sciences, University of Reading; 2 School of Applied Psychology and Australian Institute of Suicide Research and Prevention, Griffith University; 3 School of Psychology and Clinical Language Sciences, University of Reading

**Keywords:** anxiety, child, identification, brief measure

## Abstract

Anxiety disorders are among the most prevalent mental health disorders experienced by children and are associated with significant negative outcomes. Only a minority of affected children, however, access professional help, and a failure to identify children with anxiety disorders presents a key barrier to treatment access. Existing child anxiety questionnaire measures are long and time consuming to complete, limiting their potential for widespread use as identification tools in community settings. We developed a brief questionnaire for parents, children, and teachers using items from the Spence Children’s Anxiety Scale (SCAS) and evaluated the new measure’s psychometric properties, capacity to discriminate between a community (*n* = 361) and clinic-referred sample (*n* = 338) of children aged 7–11, and identified optimal cut-off scores for accurate identification of preadolescent children experiencing clinically significant levels of anxiety. The findings provided support for the reliability and validity of 8-item versions of the SCAS, with the brief questionnaire scores displaying comparable internal consistency, agreement among reporters, and convergent/divergent validity to the full-length SCAS scores. The brief SCAS scores also discriminated between the community and clinic-referred samples and identified children in the clinic-referred sample with a moderate-to-good level of accuracy and acceptable sensitivity and specificity. Combining reporters improved sensitivity, but at the expense of specificity, and findings suggested parent report should be prioritized. This new brief questionnaire has potential for use in community settings as a tool to improve identification of children who are experiencing clinically significant levels of anxiety and warrant further assessment and potential support.

## Public Significance Statement

We developed and evaluated brief versions of the Spence Children’s Anxiety Scale for parents, children, and teachers. Results provide support for the potential application of this new brief questionnaire in community settings to improve identification of children with anxiety disorders.

Anxiety disorders are the most common mental health disorders experienced by children and young people (Polanczyk, Salum, Sugaya, Caye, & Rohde, 2015), with half of all lifetime anxiety disorders emerging by age 11 (Kessler, Chiu, Demler, Merikangas, & Walters, 2005). Anxiety disorders during childhood are associated with impaired academic, financial, social, and health functioning and place an individual at increased risk for continued or recurring anxiety and other mental health disorders later in life (Copeland, Angold, Shanahan, & Costello, 2014; Essau, Lewinsohn, Olaya, & Seeley, 2014). The high prevalence and significant negative outcomes associated with child anxiety disorders, coupled with the associated economic burden for society (Fineberg et al., 2013), highlight the importance of effective early intervention. However, while effective child anxiety treatments exist (James, James, Cowdrey, Soler, & Choke, 2013), only a minority of children affected by anxiety disorders access treatment (Chavira, Stein, Bailey, & Stein, 2004; Merikangas et al., 2011).

For a child to access anxiety treatment, they need to be identified as experiencing a clinically significant anxiety problem, and recent reviews of barriers to child mental health treatment illustrate the difficulties that both parents (Reardon et al., 2017) and primary care practitioners (O’Brien, Harvey, Howse, Reardon, & Creswell, 2016) face identifying mental health difficulties in children. In particular, parents report that difficulties recognizing a child’s mental health problem, and difficulties recognizing the severity and impact of a problem are barriers to help-seeking (Reardon et al., 2017); primary care practitioners report that a lack of confidence in identification, time restrictions, and a lack of tools and resources hinders recognition of child mental health problems (O’Brien, et al., 2016). The availability of accurate identification tools could help overcome these barriers and improve identification of children with anxiety disorders in community settings.

A number of questionnaires designed to assess anxiety in children exist, typically consisting of corresponding child and parent report questionnaires (e.g., Spence Children’s Anxiety Scale, SCAS; Revised Children's Anxiety and Depression Scale, RCADS [a derivative of the SCAS]; Screen for Child Anxiety Related Disorders, SCARED; Multidimensional Anxiety Scale for Children, MASC 2). As a potential tool for identifying children with clinically significant levels of anxiety, the SCAS, has the following strengths: (a) it was designed specifically to assess symptoms of *Diagnostic and Statistical Manual of Mental Disorders* (4th ed., *DSM–IV*) anxiety disorders in children, (b) it was developed within a community (rather than clinical) population, and (c) it is available free of charge. Indeed, both the child and parent-report versions of the SCAS (SCAS-C/P) have been well evaluated in community and clinical samples of children and young people with evidence to support their internal consistency, test–retest reliability, convergent and divergent validity, and capacity to discriminate between children with anxiety disorders versus community samples (e.g., Arendt, Hougaard, & Thastum, 2014; DeSousa et al., 2014; Nauta et al., 2004; Orgilés, Fernández-Martínez, Guillén-Riquelme, Espada, & Essau, 2016; Spence, Barrett, & Turner, 2003; Whiteside & Brown, 2008). A few studies have also examined the capacity of the SCAS-C/P and their subscales to identify specific anxiety diagnoses (Brown-Jacobsen, Wallace, & Whiteside, 2011; Whiteside, Gryczkowski, Biggs, Fagen, & Owusu, 2012) and to discriminate between those with anxiety versus nonanxiety psychiatric diagnoses (Olofsdotter, Sonnby, Vadlin, Furmark, & Nilsson, 2016). However, data relating to optimal cut-off scores on the SCAS-C/P that maximize sensitivity (correct classification of children with anxiety disorders) and specificity (correct classification children without anxiety disorders) among preadolescent children are not currently available. Furthermore, the SCAS-C/P consists of 38 items and as such is time consuming to complete, limiting its potential for widespread application as a tool for identifying children with clinically significant levels of anxiety in community settings. Validated brief questionnaires designed to identify anxiety and depressive disorders in adults are widely used in primary care settings (GAD-7; Spitzer, Kroenke, Williams, & Löwe, 2006; PHQ-9; Kroenke, Spitzer, & Williams, 2001), but corresponding, well-evaluated, brief questionnaires to assess anxiety in children are yet to be developed despite their clear utility in both primary care and school settings. Shorter versions of the RCADS have been developed (including a 20-item anxiety scale; Muris, Meesters, & Schouten, 2002) and a 15-item anxiety scale (Ebesutani et al., 2012), but these are not as brief as the adult equivalents and may be too long for routine use in, for example, primary care settings where time constraints are a particular concern (Klinkman, 1997; O’Brien et al., 2016). Primary care appointments are short, typically lasting less than 10 min (Hobbs et al., 2016), making questionnaire length and completion time key determinants of the acceptability of identification tools (Kroenke, Monahan, & Kean, 2015; Mitchell & Coyne, 2007). Indeed, brevity has been prioritized in the development of adult mental health screening tools, with a focus on minimizing the number of items required for accurate identification (Spitzer et al., 2006) and ensuring completion time of less than 5 min (Mitchell & Coyne, 2007), with typically fewer than 10 items (Kroenke et al., 2015). Moreover, there is evidence to support the potential role of teachers in identifying mental health problems in children and the benefit of adopting a multiple informant approach to child mental health screening, particularly where difficulties may be context-dependent (De Los Reyes, et al., 2015; Goodman, Ford, Simmons, Gatward, & Meltzer, 2000). The evidence base surrounding teacher-report questionnaires designed to assess symptoms of anxiety disorders in children is, however, limited. There have been promising findings from an initial evaluation of a 16-item teacher questionnaire that includes some SCAS items together with new items (Lyneham, Street, Abbott, & Rapee, 2008). However, similar to primary care settings, questionnaire length and completion time are key determinants of the acceptability of mental health screening questionnaires in school settings (Levitt, Saka, Romanelli, & Hoagwood, 2007), indicating the need to prioritize brevity and minimize the number of items in teacher-report questionnaires. A brief teacher-report questionnaire (with <10 items), including data relating to optimal cut-off scores to identify children with clinically significant levels of anxiety in children, is not currently available.

The aims of this study were to develop a brief questionnaire (child, parent, and teacher versions) designed to assess symptoms of *DSM* child anxiety disorders using items from the SCAS-C/P among 7- to 11-year-olds; and (a) to evaluate the reliability and validity of the brief SCAS scores (child, parent, teacher versions) in a community and clinic-referred sample of children; (b) to establish the capacity of the brief SCAS scores (child, parent, teacher versions) to discriminate between a community and clinic-referred sample of clinically anxious children, including the relative contribution of each reporter and the optimal combination of reporters; and (c) to identify optimal cut-off scores on the brief SCAS (child, parent, teacher versions) for accurate identification of children with elevated anxiety symptoms for whom further clinical investigation is warranted.

## Method

### Participants

Participants included a community sample and a clinic-referred sample of children and their parent/carer and class teacher. Characteristics of each sample are detailed in Table 1.[Table-anchor tbl1]

The community sample were recruited as part of a wider study of parental perceived barriers and facilitators to seeking and accessing professional help for anxiety disorders in children (see Reardon, Harvey, Young, O’Brien, & Creswell (2018) for full study details). The study was approved by the University of Reading Research Ethics Committee (UREC 15/04). As displayed in Table 1, this sample consisted of 361 children (192 girls, 169 boys) recruited from 10 primary/junior schools in England. Children were aged 7–11 years (mean age = 9.50, *SD* = 1.09), and 46.5% were from families classed as “higher/professional.” Details of the number of parents, children, and teachers who completed the SCAS adequately (>75% items complete) are provided in Table 1.

The clinic-referred sample consisted of 338 children (170 girls, 168 boys) with a primary anxiety disorder recruited as part of two randomized controlled trials (RCTs) conducted within the University of Reading. The trials were approved by Berkshire Research Ethics Committee (reference: 07/H0505/157 and 07/H0505/156) and the University of Reading Research Ethics Committee. All 338 children were referrals to the Berkshire Child Anxiety Clinic, and 175 participated in an RCT comparing child cognitive– behavioral therapy (CCBT) alone, with CCBT supplemented by either cognitive– behavioral therapy (CBT) to target maternal anxiety or an intervention to target mother– child interactions (Creswell et al., 2015); and 163 participated in an RCT comparing two guided parent delivered CBT groups with a wait-list control (Thirlwall et al., 2013). Full details of the recruitment procedure for these trials are reported elsewhere (Creswell et al., 2015; Thirlwall et al., 2013), and children were aged 7–11 years at the time of the pretreatment assessment.

Differences between the demographic profiles of the two samples were examined. There was no significant difference between the samples on gender (χ^2^ = .71, *p* = .40). The community and clinic-referred samples did differ significantly on child age (mean age = 9.50 and 9.70 years, respectively); *t*(674) = 2.11, *p* = .04, and socioeconomic status (frequencies higher/professional; χ^2^ = 10.79, *p* = .001, *d* = .27); however, in the case of age, this reflected a negligible effect size (*d* = .16).^1^

### Procedure

#### Community sample

Primary and junior schools from different geographic locations in England were approached and invited to take part in the study. Recruited schools distributed study materials to all parents/carers of children in United Kingdom school years 3 to 6 (aged 7–11 years); and parents/carers were asked to provide consent for their child to participate in the study, and to complete questionnaire measures (SCAS-P and SDQ-P). Consent was obtained from 361 (16.2%) of the 2,223 parents/carers invited to take part in the study. Corresponding questionnaires (SCAS-C and SDQ-C) were administered by a member of the research team with the children during a visit to the school, and class teachers were asked to complete corresponding questionnaires about the children whose parent provided consent (SCAS-T and SDQ-T).

#### Clinic-referred sample

Children in both trials were assessed prior to randomization and treatment group allocation, and this data was used in the current study. As part of this assessment, parents and children completed the Anxiety Disorders Interview Schedule-Child and Parent Interviews (ADIS-IV-C/P) and the SCAS-C/P and SDQ-C/P, and teachers were asked to complete the SCAS-T and SDQ-T.

### Measures

#### Spence Children’s Anxiety Scale (SCAS-C, SCAS-P, SCAS-T)

The SCAS is a 38-item questionnaire designed to assess a child’s anxiety symptoms, and includes corresponding child (SCAS-C; Spence, 1997, 1998) and parent report (SCAS-P; Nauta et al., 2004) versions. Items address symptoms of *DSM–IV* anxiety disorders, including separation anxiety, generalized anxiety, social phobia, obsessive–compulsive behaviors, panic and agoraphobia, and physical injuries fears. Items are rated on a 4-point scale (0–3; never–always) and total scores reflect the sum of responses to the 38 items. SCAS-C/P total scores were calculated if >75% of items were complete, and in cases with missing data, the average total score using completed items was calculated. The reliability and validity of the SCAS-C/P scores have been reported in community and clinical samples (Arendt et al., 2014; Nauta et al., 2004; Whiteside & Brown, 2008).

A teacher report version of the SCAS (SCAS-T) was developed by the research team, and includes 30 items from the SCAS rated on the same 4-point rating scale. Items were reworded to account for the change in reporter and eight SCAS-C/P items relating to symptoms that teachers would not be able to observe were omitted (e.g., items relating to sleep, animal fears). No new items were added. SCAS-T total scores reflect the sum of responses to the 30 items, and were calculated if >75% of items were complete, and in cases with missing data, the average across completed items was used.

#### Strengths and Difficulties Questionnaires (SDQ-C; SDQ-P/T)

The SDQ (Goodman, 1997) provides a broad-based measure of a child’s emotional and behavioral difficulties. The child (SDQ-C) and parent/teacher (SDQ-P/T) report versions include corresponding items addressing a child’s emotional symptoms (five items), peer relationship problems (five items), conduct problems (five items), and hyperactivity/inattention (five items), with strong evidence in support of its psychometric properties both in community (Goodman et al., 2000) and clinic-referred samples (Goodman, Renfrew, & Mullick, 2000). In this study the SDQ-emotional problems scale, internalizing problems scale (emotional + peer relationship problems), conduct problems scale, and externalizing problems scale (conduct + hyperactivity/inattention) were used to examine the convergent and divergent validity of scores on the new brief anxiety measure. The internal consistency for the SDQ scale scores were acceptable-good in the current samples (SDQ-emotional problems scale, child α = .76, parent α = .84, teacher α = .85; SDQ-internalizing problems scale, child α = .76, parent α = .82, teacher α = .82; SDQ-conduct problems scale, child α = .60, parent α = .65, teacher α = .70; SDQ-externalizing problems scale, child α = .74, parent α = .82, teacher α = .85).

#### ADIS-C/P

The ADIS-C/P was administered with the clinic-referred sample to assess the child’s diagnostic status, including the assessment of *DSM–IV* anxiety, mood and externalizing disorders. The reliability and validity of the ADIS has been widely reported (Silverman, Saavedra, & Pina, 2001). As per the standard guidelines, overall diagnoses and Clinical Severity Ratings (CSRs; 4–8) were assigned if the child met diagnostic criteria based on either the child or parent report, and the higher of the two CSRs was assigned. The disorder with the highest CSR was assigned as the primary disorder. Assessors were psychology graduates in both trials, and all assessments were discussed with a consensus team for at least the first 20 interviews for each assessor, at which stage the assessor’s reliability was checked (minimum κ = .85). After this point, at least one in six interviews were discussed with a consensus team; and overall reliability within the assessment team in both trials was excellent (child-report diagnosis: κ = 0.98; CSR: intraclass correlation [ICC] = 0.98–0.99; parent-report diagnosis: κ = 0.98; CSR: ICC = 0.97–0.99).

### Data Analytic Approach

#### Development of Brief Versions of the SCAS

The following procedure was used to develop a brief version of the SCAS (parent, child and teacher report versions). (1) The functioning of SCAS-P/C/T items was examined in the two samples combined, including item-differential functioning (Univariate Logistic Regression; SCAS item score = independent variable, sample = dependent variable) and item-total correlations (Pearson’s *r* correlation coefficient). In addition, because the SCAS-T is a new measure, response rates for each item were examined and items with a very high proportion (>97%) of “never” responses in the community sample (suggesting the item is not appropriate for teachers) were not considered for inclusion in the brief questionnaire. (2) Alternative potential versions of brief parent/child/teacher questionnaires (including varying combinations of 6–10 items) were developed, prioritizing items that showed significant prediction of community/clinic-referred sample membership (odds ratio >2.00), with at least moderate item-total correlation (*r* > .50). Content of items was also considered to: (a) minimize overlap between items, (b) include items that address symptoms of a range of types of anxiety disorders (excluding the obsessive–compulsive items to reflect the change in the *Diagnostic and Statistical Manual of Mental Disorders* (5th ed., *DSM-5*) classification of anxiety disorders), and (c) where possible to maximize the number of common items across the parent/child/teacher questionnaire. (3) receiver operating characteristics (ROC) analyses were used to compare the capacity of alternative potential versions of parent/child/teacher brief questionnaires to identify children with anxiety disorders (i.e., the clinic-referred sample). The area under the curve (AUC) statistic was examined, and as per previous studies examining the AUC statistic associated with anxiety screening tools (van Gastel & Ferdinand, 2008; Villabø, Gere, Torgersen, March, & Kendall, 2012), AUC = .70 was taken as the minimum threshold to indicate that the measure was moderately accurate at identifying children in the clinic-referred sample. The sensitivity and specificity values for alternative cut-off scores were also examined. Given the purpose of the measure is to identify children with anxiety disorders, sensitivity was prioritized, with the optimal cut-off score reflecting sensitivity values >.80 and specificity >.70. In cases where it was not possible to achieve sensitivity/specificity values of .80/.70, respectively, cut-off scores with lower sensitivity/specificity values (>.60) were considered (where possible selecting cut-off values with sensitivity >.70). Findings from the ROC analyses for the brief parent/child/teacher SCAS with the optimal capacity to identify children with anxiety disorders are reported.

#### Evaluation of the brief questionnaires

Total scores on the optimal brief versions of the parent/child/teacher SCAS were calculated using the same procedure to deal with missing data as detailed previously for the full length SCAS (total scores reflect the sum of responses to all included items). The following psychometric properties of scores on the optimal brief versions of the parent/child/teacher SCAS were examined in each sample and compared with scores on the full length SCAS-P/C/T: (a) internal consistency (Cronbach’s alpha coefficients); (b) agreement between reporters (Pearson’s *r* correlation coefficients); (c) convergent and divergent validity (Pearson’s *r* correlation coefficient between full/brief SCAS scores and SDQ internalizing/emotional/externalizing/conduct scale scores). The capacity of the optimal brief parent/child/teacher SCAS scores to discriminate between children in the clinic-referred sample and children in the community sample was examined for the total sample, and for gender groups using (a) independent sample *t* tests (and Cohen’s *d*), and (b) ROC analyses (as detailed previously, examining both the AUC and the sensitivity/specificity values associated with optimal cut-off scores on the parent/child/teacher brief SCAS). To compare the functioning of the brief SCAS with the full-length SCAS, the capacity of the full-length SCAS to discriminate between the two samples was also analyzed. A series of logistic regressions were used to examine the contribution of each reporter (parent, child, teacher), to determine whether using multiple informants improves the capacity of the brief SCAS scores to identify children in the clinic-referred sample. Using optimal cut-off scores identified in the ROC analyses, the sensitivity and specificity values associated with each combination of reporters (parent + child, parent + teacher, teacher + child, parent + child + teacher) were examined. For each combination of reporters, the sensitivity value reflected the proportion of children in the clinic-referred sample who scored above the optimal cut-off score based one at least one of reporter; and specificity value reflected the proportion of children in the community sample who scored below the optimal cut-off for each reporter. Gender differences on total scores on the brief (and full-length SCAS) within each sample (independent samples *t* tests) were also examined.

Because the sample sizes were large (>330 in each sample), a conservative *p* value (*p* < .01) was used to indicate a statistical significance. All analyses were conducted using IBM SPSS (Version 21).

## Results

### Development of Brief Parent/Child/Teacher SCAS

Rank-ordered item-total correlations for SCAS-P items in the two samples combined, together with item differential functioning statistics are detailed in Table 2. Items selected for the brief parent questionnaire (SCAS-P-8) are also displayed in Table 2, with item-total correlations ranging from .56–.70, and all items were significant predictors of sample (*p* < .001), with higher scores among the clinic-referred sample (odds ratio = 1.94–6.87). Because obsessive compulsive disorder is no longer classed as an anxiety disorder within *DSM-5*, two items addressing obsessive–compulsive behaviors (Item 17 and 36), with strong item-total correlations were not considered for inclusion in the brief measure. The selected items addressed generalized anxiety (three items), social anxiety (two items), separation anxiety (two items), and panic/agoraphobia (one item).[Table-anchor tbl2]

Table 3 details the SCAS-C rank-ordered item-total correlations, and associated differential functioning statistics associated with each item, together with items selected for the brief child questionnaire (SCAS-C-8). The predictive values associated with SCAS-C item scores were notably smaller than for SCAS-P items. Item-total correlations for SCAS-C-8 items ranged from .52–.68, and selected items significantly predicted the sample (*p* < .001), with higher item scores among the clinic-referred sample (odds ratio = 1.39–2.00). As with the SCAS-P-8, items addressing obsessive–compulsive behavior were not considered for inclusion in SCAS-C-8 (Items 41 and 19). The two social anxiety items from the SCAS-P-8 (Items 9 and 29) were not selected for inclusion in the SCAS-C-8 because neither were significant predictors of community/clinic-referred sample, and including these items reduced the overall capacity of the brief child questionnaires to discriminate between the two groups. Item 22 (“I worry something bad will happen to me”) and Item 32 (“all of a sudden I feel really scared for no reason at all”) from the SCAS-P-8 were also less strongly associated with the clinic-referred sample based on child-report (odds ratio = 1.29 and 1.31, respectively) than alternative SCAS-C items and were therefore also not selected for inclusion in the SCAS-C-8. Final selected SCAS-C-8 items address generalized anxiety (two items), separation anxiety (4 items), and panic/agoraphobia (two items), and four of these items appear on the SCAS-P-8.[Table-anchor tbl3]

Table 4 displays the rank-ordered item-total correlations and associated item functioning for 20 items included in the SCAS-T. Ten SCAS-T items were not considered for inclusion in the brief measure because they were associated with very low response rates (seven items; >97% “never” response in the community sample) or addressed obsessive–compulsive behavior (three items). Items selected for the SCAS-T-8 are identified in Table 4, with item-total correlations ranging from .56–.75, and all items were significant predictors of sample (*p* < .001), with higher scores among the clinic-referred sample (odds ratio = 1.89–5.35). Three SCAS-T-8 items appear on both the SCAS-P-8 and SCAS-C-8 (Item 1, Item 6, Item 12), and a further three items appear on the SCAS-P-8 (Item 7, Item 16, Item 22). The social anxiety item addressing worries about school (“worries that he or she will do badly at school”) had the second highest item-total correlation (.74) among SCAS-T items and, with its focus on school, appears particularly relevant to teachers so was selected to replace “worries what others think” from the SCAS-P-8. Item 15 (“suddenly starts to tremble or shake”) from the SCAS-C-8 was also selected for the brief teacher questionnaire as scores on this item were more strongly associated with sample than the SCAS-P-8/SCAS-C-8 item “feels afraid” (odds ratio = 3.67 compared with 1.58). Selected SCAS-T-8 items address generalized anxiety (two items), social anxiety (two items), separation anxiety (two items), and panic/agoraphobia (two items).[Table-anchor tbl4]

### Evaluation of SCAS-P-8, SCAS-C-8, and SCAS-T-8

#### Internal consistency

Internal consistency for the brief and full SCAS within each sample are provided in online supplement 1. Cronbach’s alpha coefficients for the brief questionnaires ranged from .80–.84 in the community sample, and .73–.85 in the clinic-referred sample, indicating items have an acceptable-to-good level of internal consistency.

#### Agreement between reporters

Agreement between reporters within each sample are provided in online supplement 2, indicating similar levels of agreement on the brief SCAS as the full SCAS. For the brief questionnaires, parent–child agreement was the highest (community sample, *r* = .40, *p* < .001; clinic-referred sample, *r* = .34, *p* < .001) and teacher–child agreement the lowest (community sample, *r* = .25, *p* < .001, clinic-referred sample, *r* = .05, *p* = .46).

#### Convergent and divergent validity

Convergent and divergent validity indices for the brief and full SCAS scores within each sample are provided in online supplement 3. Similar patterns were observed for the brief SCAS scores as for the full SCAS scores, with significantly higher correlations between the brief parent/child/teacher SCAS scores and the SDQ-emotional problems scale scores (*r* = .62–.76) and the SDQ-internalizing scale scores (*r* = .58-.70), than between the brief parent/child/teacher SCAS scores and the SDQ-conduct problems scale scores (*r* = .08-.32) and SDQ-externalizing problems scale scores (*r* = .10–.34; *z* = 4.91–9.16, *p* < .0001).

#### Discriminating between community sample and clinic-referred sample

##### Sample differences on questionnaires

As displayed in Table 5, mean SCAS-P-8 scores were significantly higher in the clinic-referred sample than in the community sample, *t*(671) = 19.51, *p* < .001, with a large effect size (*d* = 1.49). This finding was replicated among gender-differentiated groups (*d* = 1.50–1.51), and similar sample differences were observed for the full SCAS-P scores (*d* = 1.39–1.54).[Table-anchor tbl5]

As displayed in Table 5, Mean SCAS-C-8 scores were also significantly higher in the clinic-referred sample than in the community sample, *t*(647) = 8.73, *p* < .001, with a medium effect size (total sample, *d* = 0.69; boys, *d* = 0.77; girls, *d* = 0.67). Sample differences for the full child SCAS scores represented small-medium effect sizes (total sample, *d* = 0.41; boys, *d* = 0.51; girls; *d* = 0.38).

Sample differences on the teacher questionnaires are also displayed in Table 5. Mean SCAS-T-8 scores were significantly higher in the clinic-referred sample than in the community sample, *t*(568) = 12.43, *p* < .001, with a large effect size (*d* = 1.01). This finding was replicated among gender-differentiated groups (boys, *d* = 0.93; girls, *d* = 1.10), and similar sample differences were observed for the SCAS-T-20 scores (*d* = 0.86–1.01).

##### ROC analyses

As displayed in Table 6, the SCAS-P-8 was able to accurately identify children in the clinic-referred sample with an AUC of .86, and using an optimal cut-off score of 7.5, achieved .85 sensitivity and .75 specificity overall (with sensitivity/specificity values of .81/.79 for boys; and .89/.71 for girls). Corresponding sensitivity/specificity values for optimal cut-off scores on the full SCAS-P were 82/.78 (boys, .83/.80; girls, .82/.77).[Table-anchor tbl6]

The SCAS-C-8 also achieved an AUC >.70, both in the total sample and the gender differentiated groups (boys, .74; girls, .70). ROC analyses examining the SCAS-C-8 in the total sample indicated that the optimal cut-off score was 6.5, achieving a sensitivity value of .67, and specificity of .64 (it was not possible to achieve sensitivity >.70, with specificity >.60 for the total sample). The ROC analyses among the gender differentiated groups, however, indicated that the optimal cut-off scores among boys was 5.5, and among girls was 7.5, with respective sensitivity/specificity values of .73/.70, and .64/.63. The full child SCAS failed to achieve an AUC >.70 in the total sample or among gender differentiated groups, and the optimal cut-off scores on full child SCAS achieved similar sensitivity to the SCAS-C-8 (boys, .71; girls, .61), but with lower specificity (boys, .61; girls, .55).

The SCAS-T-8 achieved an AUC of .76, and the optimal cut-off score of 4.5 in the total sample was associated with a sensitivity value of .70, and specificity of .73. Analyses among gender differentiated groups indicated the optimal cut-off score on the SCAS-T-8 among boys was 3.5 (sensitivity/specificity, .74/.64), and among girls was 4.5 (sensitivity/specificity, .73/.69). Optimal cut-off scores on the SCAS-T-20 achieved sensitivity/specificity values of .71/.71 among boys, and .74/.64 among girls.

#### Using multiple reporters and the contribution of each reporter

Findings from the series of Logistic Regressions using different combinations of the SCAS-P-8, SCAS-C-8 and SCAS-T-8 scores to predict whether the child was in the community or clinic-referred sample are displayed in Table 7. Among the models including two reporters, using parent report (SCAS-P-8) and teacher report (SCAS-T-8) explained the most variance (Nagelkerk, .54, Cox & Snell, .40); and scores on both the SCAS-P-8 and SCAS-T-8 were uniquely associated with sample (odds ratio, 1.40 and 1.18, respectively). Replacing the teacher report (SCAS-T-8) with the child report (SCAS-C-8) only slightly reduced the total amount of variance explained (Nagelkerk, .47, Cox & Snell, .35), although in this parent + child model, the SCAS-C-8 score was not significantly associated with the sample. Using teacher report (SCAS-T-8) and child report (SCAS-C-8) explained the least variance of all of the models (Nagelkerk, .33, Cox & Snell, .24), but both the SCAS-T-8 score and SCAS-C-8 score made small significant contributions (odds ratio = 1.28 and 1.12, respectively). In the model including all three reporters, higher scores on the SCAS-P-8 best predicted whether participants were in the community or clinic-referred sample (odds ratio = 1.39), and the SCAS-T-8 score also made a significant unique contribution (odds ratio = 1.17), but the SCAS-C-8 did not (odds ratio = 1.02).[Table-anchor tbl7]

As displayed in online supplement 4, the brief SCAS scores accurately identified >89% of children in the clinic-referred sample when multiple reporters were used, with the highest sensitivity achieved when all three brief questionnaires are combined (.97), and lowest when teacher and child report are combined (.89). The brief SCAS specificity was reduced when multiple reporters were combined; ranging from .54 (parent + teacher and parent + child) to .42 (parent + teacher + child) based on the optimal cut-off points identified in Table 6.

#### Gender differences

Gender means for the brief and full length SCAS scores are displayed in Online Supplement 5. Significant gender effects were found for the SCAS-C-8, with significantly higher scores among girls than boys both in the community sample, *t*(322) = 3.78, *p* < .001, *d* = .42 and clinic-referred sample, *t*(323) = 2.90, *p* < .001, *d* = .32; and this same pattern was observed on the full length SCAS-C. No significant gender differences were found on either the SCAS-P-8 scores or SCAS-T-8 scores; although scores on the full length SCAS-P were significantly higher among girls than boys within the community sample, *t*(355) = 2.91, *p* < .001, *d* = .31.

## Discussion

In this study, we developed a brief questionnaire (parent, child, and teacher-report versions) designed to assess symptoms of *DSM-5* anxiety disorders. Each version of the brief questionnaire (SCAS-P-8, SCAS-T-8, SCAS-C-8) includes eight SCAS items. Item functioning and the content of items were considered to select items for inclusion in the brief questionnaire. Item functioning varied across reporters, and to maximize performance of each version of the questionnaire, the selected items varied across reporters (with three common items across the SCAS-P-8, SCAS-T-8, and SCAS-C-8). Each version of the brief questionnaire includes items that address generalized anxiety, separation anxiety, and panic/agoraphobia; and the SCAS-P-8 and SCAS-T-8 also includes items that address social anxiety.

The findings provide support for the reliability and validity of the SCAS-P-8, SCAS-C-8, and SCAS-T-8 scores in a community and clinical sample of children with anxiety disorders. In line with previous studies of the full length SCAS (Arendt et al., 2014; Nauta et al., 2004; Spence, 1998; Whiteside & Brown, 2008), the brief questionnaire scores displayed acceptable to good internal consistency in both samples, although not as strong as the full-length SCAS scores. Similar levels of agreement among reporters were observed for the brief SCAS scores as the full-length SCAS scores, with highest agreement between parent and child and lowest between teacher and child. In relation to convergent and divergent validity, the brief questionnaire also displayed similar patterns to the full-length SCAS, with the SCAS-P-8, SCAS-C-8, and SCAS-T-8 scores each significantly correlated with the SDQ-internalizing and emotional problems scale scores, and weakly correlated with SDQ-externalizing and conduct problems scale scores.

The findings also illustrated the capacity of the SCAS-P-8, SCAS-C-8, and SCAS-T-8 scores to discriminate between the clinic-referred sample and the community sample. As previously reported for the full length SCAS (Arendt et al., 2014; Nauta et al., 2004; Spence, 1998; Whiteside & Brown, 2008), scores on each version of the brief questionnaire were significantly higher among the clinic-referred sample than the community sample. ROC analyses also indicated that the SCAS-P-8, SCAS-C-8 and SCAS-T-8 scores were each able to identify children in the clinic-referred sample with at least a moderate level of accuracy (AUC > .70) with an acceptable level of sensitivity and specificity. The SCAS-P-8 score identified children in the clinic-referred sample with a good level of accuracy (AUC = .86), and the optimal cut-off score of 7.5, achieved sensitivity/specificity values > .80/.70, respectively (.85/.75 for the total sample; .81/.79 for boys; and .89/.71 for girls). Optimal cut-off scores on the SCAS-C-8 (5.5 for boys; 7.5 for girls) achieved sensitivity/specificity values >. 70 among boys (.73/.70), and >.60 among girls (.64/.63); and optimal cut-off scores on the SCAS-T-8 (3.5 for boys; 4.5 for girls) achieved sensitivity/specificity values > .70/.60, respectively (.74/.64 for boys; .73/.69 for girls). SCAS-C-8 total scores were significantly higher among girls than boys, thus accounting for gender differentiated optimal cut-off scores; although interestingly, there were not significant difference between boys and girls on the SCAS-T-8 (or SCAS-P-8), despite the gender differentiated optimal cut-off scores on the brief teacher questionnaire.

Encouragingly, the ROC analyses also indicted that the SCAS-P-8 and SCAS-T-8 scores were able to identify children in the clinic-referred sample with a similar level of accuracy as the full length SCAS scores, suggesting reducing the SCAS-P/T to eight items does not reduce its capacity to discriminate clinically anxious children from children in the community. Furthermore, the SCAS-C-8 score displayed a higher level of accuracy than the full-length SCAS score which did not achieve an AUC > .70 in the total sample or among gender groups. The optimal cut-off scores on the full length SCAS-C were also associated with lower specificity values (.55–.61) than the SCAS-C-8, thus illustrating the advantage of using a subset of optimally functioning SCAS-C items. It is interesting that the capacity of the individual SCAS-C items to discriminate between the community and clinic-referred sample was notably lower than that for the SCAS-P items and the SCAS-T items; and this was particularly marked for social anxiety items, suggesting that it may be difficult for preadolescent children to differentiate between developmentally appropriate and clinically significant levels of social anxiety.

Findings indicated some benefit to adopting a multiinformant approach, suggesting that a combined parent plus teacher score provides the optimal combination of reporters for the detection of children with an anxiety disorder, although parent report should be prioritized above either child or teacher. It is interesting that previous studies examining the use of child and parent report to identify particular anxiety disorders among clinical samples suggest each reporter does provide unique information (Villabø et al., 2012; Wei et al., 2014), but that there may be variation in the capacity of each reporter to identify particular types of anxiety disorders, and with different patterns among children versus adolescents (Wei et al., 2014). Indeed, while our findings suggest parent report should be prioritized above child (or teacher) report to identify preadolescent children with clinically significant levels of anxiety, this may not extend to older children and adolescents, or to situations where the aim is to identify particular anxiety disorders within a clinical population. Moreover, the stronger capacity for the parent report questionnaire to identify children in the clinic-referred sample than either the child or teacher report questionnaire may, however, at least in part reflect a dominant influence of parent report in the diagnostic assessment. Diagnostic outcomes derived from the ADIS among preadolescent children show higher levels of agreement with parent report than child report (Evans, Thirlwall, Cooper, & Creswell, 2017; Grills & Ollendick, 2003); therefore, it may not be surprising that the parent report questionnaire score is the best predictor of sample in this study. Using multiple reporters improved the capacity of the brief questionnaire to correctly identify children in the clinic-referred sample (increased sensitivity), but this advantage would need to be weighed up against the reduced specificity associated with using multiple reporters unless alternative cut-off points are used to optimize specificity and sensitivity when multiple informants are used.

### Implications

This new brief anxiety questionnaire has potential for use in schools and primary care settings as a tool to improve identification of children who are experiencing high levels of anxiety and for whom a clinical diagnostic assessment may be warranted. With only eight items, the questionnaire is very quick to administer, providing a more time efficient alternative to existing questionnaires (e.g., the 38-item SCAS, the 47-item RCADS, the adapted 15-item and 20-item RCADS anxiety scales). Moreover, the availability of parent, child, and teacher-report versions maximizes potential application across situations where only one particular reporter may be available (e.g., teachers in schools), and where multiple reporters may be available (e.g., parents and children in a primary care settings). The GAD-7 is widely used in primary care settings to aid identification of anxiety disorders in adults, and is recommended as an initial screening tool where an anxiety disorder is suspected to determine if further assessment is required (National Institute for Clinical Excellence, 2011). This new brief questionnaire provides an equivalent tool for use with children, parents, and teachers to aid identification of potential cases of clinically significant levels of anxiety and to help determine whether further assessment and support is needed.

### Limitations

It is important to note several limitations associated with this study. The study examined the capacity of the new brief questionnaire scores to discriminate between a community sample and a clinic-referred sample of children who met criteria for an anxiety disorder. Diagnostic assessments, however, were not administered with the community sample, and given the prevalence rates of anxiety disorders, it can be assumed that the community sample also included some children who would have met criteria for an anxiety disorder. This would have reduced the capacity of the brief questionnaire scores to discriminate between the two samples. Future research should examine the capacity of the measure to discriminate between clinically anxious and nonanxious children where this status has been established through a diagnostic interview. It is also likely that there was a degree of participation bias in the community sample given that parents were informed that the wider study was also examining barriers to accessing anxiety treatment, and the response rate was relatively low (16.2%), thus those who were concerned about their child’s anxiety may have been more likely to take part in the study. As a result, the community sample may have included more anxious children than the general population. In fact, among boys the mean score on the full SCAS-C (26.12) and full SCAS-P (16.23) were similar to published norms (26.65 and 16.0, respectively; available at www.scaswebsite.com), but among girls the mean scores (SCAS-C, 36.18; SCAS-P, 20.13) were higher than reported elsewhere (34.02 and 15.9, respectively; available at www.scaswebsite.com), indicating that the community sample may have included more anxious girls than the general population. Thus, again the results from the present study may have underestimated the capacity of the brief questionnaire scores to discriminate between children with and without anxiety disorders.

It is also important to acknowledge that the proportion of teachers who completed SCAS questionnaires in the clinic-referred sample (63%) was relatively low compared with the proportion of teachers in the community sample (94%), and the proportion of parents and children in both samples (>89%). It is likely that the lower return rate among teachers in the clinic-referred sample is because of methodological differences in questionnaire administration across reporters and samples. In the clinic-referred sample, teacher questionnaires were administered by post, whereas children and parents completed questionnaires as part of face-to-face assessment sessions; and the community sample were recruited through schools as part of a wider study that involved researchers visiting schools to administer questionnaires. It will be important for future studies to consider methods that maximize teacher response rates among samples recruited in clinical settings.

This study also examined a number of other reliability and validity indices (internal consistency, agreement among reporters, convergent/divergent validity), but it will be important for future evaluations of the new brief questionnaire to examine its test–retest reliability. Moreover, we developed and evaluated the new questionnaire in a single study in which participants completed the full version of the SCAS. Thus, further research is now needed to evaluate the new measure in an independent sample that completes the abbreviated form the SCAS.

It is also important to note that this new brief questionnaire is designed to identify children with an anxiety disorder, but it does not include a sufficient number of items addressing any particular anxiety disorder to provide detailed information about specific anxiety disorders. As such this measure should be considered an initial tool to identify children who have elevated symptoms of anxiety, and for whom a more in-depth assessment is needed. This issue also applies to full-length anxiety questionnaires for children and youth. McLeod, Jensen-Doss, Wheat, and Becker (2013) cautioned against using anxiety rating scales, both general and multidimensional, as stand-alone diagnostic instruments, but noted their value in screening to identify children who warrant further assessment. Also, although the items address a range of types of anxiety, no items specifically address selective mutism or specific phobias, and the SCAS-C-8 items do not ask about social anxiety because these items did not discriminate between the clinical and nonclinical groups (although 64% of the clinical sample had social anxiety disorder). It is also noteworthy that the SCAS-P-8 does not include items that ask about physical symptoms, and the SCAS-C-8 and SCAS-T-8 both only include one such item (suddenly starts to tremble or shake), indicating that items relating to other nonphysical symptoms may be better able to identify children with clinically significant levels of anxiety. Given the capacity of the brief questionnaire scores to discriminate between children in a clinic-referred sample (who had a range of different types of anxiety disorders) and a community sample, it is likely that the items relate to symptoms that are common across anxiety disorders (e.g., general worry, feeling afraid, trouble going to school); however, the capacity of the new brief questionnaire scores to identify particular anxiety disorders is not yet known. Future research is needed to establish whether the brief questionnaire scores have greater capacity to detect some anxiety disorders (e.g., generalized anxiety disorder), than others (e.g., social anxiety disorder). Similarly, this study did not examine the capacity of the brief SCAS scores to discriminate between children with anxiety disorders and those with nonanxiety psychiatric diagnoses. The GAD-7 has reduced specificity within psychiatric samples compared with its ability to discriminate between adults with anxiety disorders and nonclinical groups (Beard & Björgvinsson, 2014; Kertz, Bigda-Peyton, & Bjorgvinsson, 2013); and it will be important for future research to examine the sensitivity/specificity associated with optimal cut-off scores on the brief SCAS in mental health service use settings.

This study provides support for this multiinformant eight-item questionnaire as a tool to identify children with anxiety disorders, together with data relating to optimal cut-off scores. Further research is needed to evaluate the ability of the brief questionnaire to identify specific anxiety disorders; and to evaluate its capacity to discriminate between children with and without any anxiety disorders in community settings in which diagnostic assessments confirm both the presence and the absence of anxiety disorders. This study focuses on identifying anxiety disorders in preadolescent children, and a corresponding brief questionnaire for adolescents should be developed and evaluated. It will also be important for future evaluations to examine the capacity of the brief questionnaire to be sensitive to changes in symptoms and functioning over time in response to treatment, and to discriminate between children with anxiety disorders and other nonanxiety psychiatric disorders.

## Supplementary Material

10.1037/pas0000570.supp

## Figures and Tables

**Table 1 tbl1:** Sample Characteristics

Characteristic	Community sample (*n* = 361)	Clinic-referred sample (*n* = 338)
Gender		
Female, *n* (%)	192 (53.2%)^b^	170 (50.3%)
Age		
*M* (*SD*)	9.50 (1.09)^c^	9.70 (1.36)
7- to 8-year-olds	126 (34.9%)	99 (29.3%)
9- to 11-year-olds	212 (58.7%)	239 (70.7%)
Socioeconomic status		
Higher/professional^a^	168 (46.5%)^d^	195 (57.7%)^e^
Other employed	133 (36.8%)	93 (27.5%)
Unemployed	22 (6.1%)	12 (3.6%)
Ethnicity		
White British, *n* (%)	277 (76.7%)^f^	287 (84.9%)^g^
SCAS-P (total score), *n* (%)	359 (99.4%)	312 (92.3%)
SCAS-P-8 (total score), *n* (%)	360 (99.7%)	313 (92.6%)
SCAS-C (total score), *n* (%)	322 (89.2%)	323 (95.6%)
SCAS-C-8 (total score), *n* (%)	324 (89.8%)	325 (96.1%)
SCAS-T (total score), *n* (%)	340 (94.2%)	214 (63.3%)
SCAS-T-20 (total score), *n* (%)	340 (94.2%)	227 (67.2%)
SCAS-T-8 (total score), *n* (%)	340 (94.2%)	230 (68.0%)
Primary anxiety diagnosis, *n* (%)		
Separation anxiety disorder		91 (26.9%)
Social anxiety disorder		64 (18.9%)
Generalized anxiety disorder		99 (29.3%)
Specific phobia		59 (17.6%)
Panic disorder with agoraphobia		3 (.9%)
Panic disorder without agoraphobia		3 (.9%)
Agoraphobia without panic disorder		9 (2.7%)
Selective mutism		1 (.3%)
Anxiety NOS		9 (2.7%)
Primary anxiety disorder, CSR *M* (*SD*)		5.59 (.79)
Presence anxiety diagnosis. *n* (%)		
Separation anxiety disorder		199 (58.9%)
Social anxiety disorder		213 (63.0%)
Generalized anxiety disorder		215 (63.6%)
Specific phobia		152 (45.0%)
Panic disorder with agoraphobia		3 (.9%)
Panic disorder without agoraphobia		7 (2.1%)
Agoraphobia without panic disorder		17 (5.0%)
Selective mutism		1 (.3%)
Anxiety NOS		12 (3.6%)
Presence of other diagnoses, *n* (%)		
OCD		9 (2.7%)
Major depressive disorder or dysthymia		42 (12.4%)
ADHD		51 (15.1%)
ODD		62 (18.3%)
*Note.* CSR = Clinical Severity Rating; Anxiety NOS = anxiety disorder not otherwise stated; OCD = obsessive compulsive disorder; PTSD = posttraumatic stress disorder; ADHD = attention-deficit/hyperactivity disorder; ODD = oppositional defiant disorder.		
^a^ Higher/professional = managers, directors, senior officials, professional occupations.		
Missing data: ^b^ *n* = 2 (.6%). ^c^ *n* = 23 (6.4%). ^d^ *n* = 38 (10.5%). ^e^ *n* = 38 (11.2%). ^f^ *n* = 22 (6.1%). ^g^ *n* = 10 (3.0%).		

**Table 2 tbl2:** Spence Children’s Anxiety Scale–Parent Report (SCAS-P) Rank-Ordered Item-Total Correlations and Item Differential Functioning

Item	Abbreviated SCAS-P item	Subscale	Item-total correlation^a^	Estimated coefficient	Wald statistic	Odds ratio
17	Can’t get bad or silly thoughts out of head	OC	.72**	1.25	131.19**	3.49
20^b^	Worries something bad happen to him/her	GA	.70**	1.10	93.98**	3.01
4^b^	Feeling afraid	GA	.70**	1.53	132.55**	4.62
36	Bothered by bad or silly thoughts	OC	.69**	1.02	92.70**	2.77
33	Worries will suddenly get scared feeling	P/A	.66**	1.38	94.59**	3.98
28^b^	All of a sudden feels really scared for no reason	P/A	.66**	1.56	92.44**	4.75
8^b^	Worries about being away from us	SEP	.66**	1.09	120.97**	2.96
11	Worries something awful happen to family	SEP	.65**	.85	85.62**	2.34
26^b^	Worries what others think of him/her	SOC	.62**	.70	57.83**	2.01
15^b^	Trouble going to school in mornings	SEP	.61**	1.20	108.23**	3.32
18	Complains heart beating really fast	GA	.61**	.92	54.82**	2.50
22	Feels shaky	GA	.61**	1.25	74.97**	3.48
3	Funny feeling in stomach	GA	.61**	.88	105.50**	2.42
38	Scared if stay away from home overnight	SEP	.61**	1.10	105.37**	2.99
6	Scared when has to take a test	SOC	.60**	.62	53.38**	1.86
9^b^	Afraid will make fool of self	SOC	.57**	.66	53.21**	1.94
10	Worries will do badly at school	SOC	.57**	.65	57.04**	1.92
1^b^	Worries about things	GA	.56**	1.93	168.36**	6.87
12	Suddenly can’t breathe	P/A	.55**	1.00	40.84**	2.71
19	Suddenly starts tremble or shake	P/A	.54**	1.32	36.13**	3.73
14	Scared if has to sleep on own	SEP	.54**	1.02	104.81**	2.77
30	Suddenly becomes dizzy or faint	P/A	.54**	1.26	43.22**	3.52
24	Special thoughts stop bad things happening	OC	.54**	.92	25.84**	2.50
32	Heart suddenly starting to beat too quickly	P/A	.53**	.94	35.62**	2.57
7	Afraid when has to use public toilets	SOC	.53**	.93	59.01**	2.53
31	Afraid when has to talk in front of class	SOC	.52**	.59	44.70**	1.80
2	Scared of the dark	PHY	.51**	.70	75.43**	2.00
21	Scared of going to doctor or dentist	PHY	.51**	.65	44.39**	1.92
5	Afraid of being on own at home	SEP	.51**	.68	83.76**	1.97
37	Has to do certain things in just right way	OC	.47**	.64	16.97**	1.89
13	Has to keep checking has done things right	OC	.47**	.70	29.21**	2.01
27	Afraid of being in crowded place	P/A	.47**	.59	24.80**	1.81
25	Scared if has to travel in car, bus or train	P/A	.43**	.97	32.25**	2.63
35	Has to do same things over and over	OC	.41**	.51	13.89**	1.67
34	Afraid of being in small closed places	P/A	.41**	.65	26.27**	1.92
29	Scared of insects or spiders	PHY	.33**	.21	6.55*	1.23
23	Scared of heights	PHY	.33**	.07	.64	1.07
16	Scared of dogs	PHY	.23**	.34	16.98**	1.41
*Note.* OC = obsessive-compulsive; GA = generalized anxiety; P/A = panic and agoraphobia; SOC = social phobia; SEP = separation anxiety; PHY = physical injuries fear.						
^a^ Use combined community sample and clinic-referred sample. ^b^ Proposed SCAS-P-8 items.						
* *p* < .01. ** *p* < .001.						

**Table 3 tbl3:** Spence Children’s Anxiety Scale–Child Report (SCAS-C) Rank Ordered Item-Total Correlations and Item Differential Functioning

Item	Abbreviated SCAS-C item	Subscale	Item-total correlation^a^	Estimated coefficient	Wald statistic	Odds ratio
22	Worry something bad will happen to me	GA	.72**	.26	9.97*	1.29
37^b^	Worry will suddenly get scared feeling	P/A	.68**	.54	32.03**	1.72
32	All of a sudden feel really scared for no reason	P/A	.67**	.27	8.47*	1.31
41	Bad or silly pictures or thoughts in mind	OC	.67**	.18	5.81	1.20
29	Worry what other people think of me	SOC	.65**	.07	.88	1.08
24	Feel shaky	GA	.65**	.18	4.26	1.19
12	Worry something awful happen to family	SEP	.64**	.12	2.73	1.13
36	Heart suddenly starts to beat too quickly	P/A	.63**	.13	1.95	1.14
9	Afraid will make fool self	SOC	.62**	.09	1.17	1.09
4^b^	Feel afraid	GA	.62**	.66	34.60**	1.94
20	Heart beats really fast	GA	.62**	.00	.00	1.00
8^b^	Worry about being away from my parents	SEP	.62**	.40	26.16**	1.49
1^b^	Worry about things	GA	.62**	.69	39.44**	2.00
19	Can’t get bad or silly thoughts out of head	OC	.61**	.28	13.23**	1.33
21^b^	Suddenly start to tremble or shake	P/A	.59**	.33	11.67**	1.39
16^b^	Trouble going to school in the mornings	SEP	.57**	.45	24.43**	1.57
39	Afraid small closed places	P/A	.57**	.11	2.24	1.12
13	Suddenly feel as if can’t breathe	P/A	.56**	.21	5.28	1.23
30	Afraid of being in crowded places	P/A	.56**	.17	3.77	1.18
35	Afraid if have to talk in front of class	SOC	.56**	.25	10.30**	1.28
42	Have to do some things in just right way	OC	.56**	.06	.50	1.06
10	Worry will do badly at schoolwork	SOC	.53**	.23	7.01*	1.26
6	Feel scared when have to take a test	SOC	.53**	.19	6.02	1.21
34	Suddenly become dizzy or faint	P/A	.52**	.08	.54	1.08
44^b^	Scared if had to stay away overnight	SEP	.52**	.43	29.59**	1.53
15^b^	Feel scared if have to sleep on own	SEP	.52**	.56	40.39**	1.76
5	Afraid to be at home alone	SEP	.52**	.35	25.53**	1.42
3	Funny feeling in stomach	GA	.52**	.28	12.76**	1.32
27	Special thoughts stop bad things happening	OC	.50**	.17	5.03	1.19
7	Feel afraid to use public bathrooms	SOC	.49**	.21	5.63	1.23
14	Keep checking that done things right	OC	.47**	.04	.25	1.04
28	Scared if have to travel in car bus or train	P/A	.46**	.29	6.73*	1.34
23	Scared of going to doctors or dentist	PHY	.45**	.24	7.95*	1.27
2	Scared of the dark	PHY	.43**	.26	13.24**	1.30
25	Scared of high places or lifts	PHY	.39**	.17	4.84	1.18
40	Have to do some things over and over	OC	.38**	.02	.07	1.02
33	Scared of insects and spiders	PHY	.35**	.03	.20	1.03
18	Scared of dogs	PHY	.20**	.19	4.93	1.21
*Note.* OC = obsessive-compulsive; GA = generalized anxiety; P/A = panic and agoraphobia; SOC = social phobia; SEP = separation anxiety; PHY = physical injuries fear.						
^a^ Use combined community sample and clinic-referred sample. ^b^ Proposed SCAS-C-8 items.						
* *p* < .01. ** *p* < .001.						

**Table 4 tbl4:** Spence Children’s Anxiety Scale–Teacher Report (SCAS-T) Rank Ordered Item-Total Correlations and Item Differential Functioning

Item	Abbreviated SCAS-T item^a^	SCAS-P/C subscale	Item-total correlation^b^	Estimated coefficient	Wald statistic	Odds ratio
16^c^	Worries something bad will happen to him/her	GA	.75**	1.30	60.31**	3.66
8^c^	Worries that he/she will do badly at school	SOC	.74**	.69	37.96**	2.00
1^c^	Worries about things	GA	.74**	.92	58.97**	2.51
4	Scared when takes test	SOC	.73**	.71	41.32**	2.03
17	Feels shaky when has a problem	GA	.72**	1.32	57.19**	3.76
22^c^	All of sudden feels scared for no reason	P/A	.71**	1.21	36.56**	3.35
7^c^	Afraid make fool self	SOC	.70**	.64	31.59**	1.89
6^c^	Worries about being away from parents	SEP	.70**	1.00	60.58**	2.73
12^c^	Trouble going to school in mornings	SEP	.69**	1.68	90.65**	5.35
26	Worries will suddenly get a scared feeling	P/A	.67**	1.19	30.05**	3.27
20	Worries what others think	SOC	.67**	.68	38.10**	1.98
9	Worries something awful will happen to family	SEP	.66**	1.02	54.09**	2.77
3	Feeling afraid	GA	.65**	.46	11.64*	1.58
24	Afraid when has to talk in front of class	SOC	.63**	.42	14.70**	1.53
21	Afraid of crowded places	P/A	.59**	.73	16.09**	2.08
15^c^	Suddenly starts to tremble or shake	P/A	.56**	1.30	22.26**	3.67
2	Tummy aches	GA	.55**	.48	15.40**	1.62
14	Complains heart beating really fast	GA	.47**	1.08	16.02**	2.94
23	Suddenly becomes dizzy or faint	P/A	.44**	.81	9.93**	2.25
10	Suddenly can’t breathe	P/A	.40**	.88	12.70**	2.40
*Note.* GA = generalised anxiety; P/A = panic and agoraphobia; SOC = social phobia; SEP = separation anxiety.						
^a^ Item functioning reported for 20 SCAS-T items considered for inclusion in brief questionnaire. ^b^ Use combined community sample and clinic-referred sample. ^c^ Proposed SCAS-T-8 items.						
* *p* < .01. ** *p* < .001.						

**Table 5 tbl5:** Differences between Community Sample and Clinic-Referred Sample on Brief SCAS and Full-Length SCAS (parent, child, Teacher Report)

	Parent report			Child report			Teacher report		
	Community Mean (*SD*)	Clinic-referred Mean (*SD*)	*t* test (Cohen’s *d*)	Community Mean (*SD*)	Clinic-referred Mean (*SD*)	*t* test (Cohen’s *d*)	Community Mean (*SD*)	Clinic-referred Mean (*SD*)	*t* test (Cohen’s *d*)
Total sample									
Brief SCAS	5.68 (3.68)	11.86 (4.53)	*t*(671) = 19.51** (*d* = 1.49)	5.97 (4.70)	9.18 (4.67)	*t*(647) = 8.73** (*d* = .69)	3.39 (2.92)	7.34 (4.67)	*t*(568) = 12.43** (*d* = 1.01)
SCAS (full)	18.28 (12.73)	39.45 (16.31)	*t*(669) = 18.85** (*d* = 1.45)	31.68 (21.02)	39.76 (18.47)	*t*(643) = 5.19** (*d* = 0.41)	6.84 (6.17)	14.61 (10.05)	*t*(565) = 11.40** (*d* = .93)
Boys									
Brief SCAS	5.33 (3.35)	11.36 (4.58)	*t*(317) = 13.47** (*d* = 1.50)	4.90 (4.47)	8.44 (4.70)	*t*(305) = 6.74** (*d* = .77)	3.19 (2.98)	6.67 (4.38)	*t*(261) = 7.69** (*d* = .93)
SCAS (full)	16.23 (10.85)	37.93 (16.64)	*t*(317) = 13.90** (*d* = 1.54)	26.12 (20.43)	35.93 (17.85)	*t*(303) = 4.48** (*d* = .51)	6.19 (6.20)	13.12 (9.53)	*t*(262) = 7.16** (*d* = .86)
Girls									
Brief SCAS	6.03 (3.91)	12.33 (4.43)	*t*(350) = 14.17** (*d* = 1.51)	6.84 (4.71)	9.93 (4.53)	*t*(340) = 6.15** (*d* = .67)	3.56 (2.86)	7.92 (4.85)	*t*(305) = 9.89** (*d* = 1.10)
SCAS (full)	20.13 (13.97)	40.90 (15.90)	*t*(348) = 13.00** (*d* = 1.39)	36.18 (20.45)	43.57 (18.33)	*t*(338) = 3.49** (*d* = .38)	7.39 (6.11)	15.96 (10.34)	*t*(301) = 9.06** (*d* = 1.01)
*Note.* Brief SCAS = SCAS-P-8/SCAS-C-8/SCAS-T-8; SCAS (full) = SCAS-P/SCAS-C/SCAS-T-20.									
** *p* < .001.									

**Table 6 tbl6:** Receiver Operating Characteristics for Parent, Teacher, and Child Questionnaires

Variable	Brief SCAS	SCAS (full)
Parent report		
Total sample		
*n* (positive; negative)	313; 360	312; 359
AUC	.86	.86
Optimal cut score	7.5	24.5
Sensitivity	.85	.82
Specificity	.75	.78
Boys		
*n* (positive; negative)	153; 166	153; 166
AUC	.86	.88
Optimal cut score	7.5	23.5
Sensitivity	.81	.83
Specificity	.79	.80
Girls		
*n* (positive; negative)	160; 192	159; 191
AUC	.86	.85
Optimal cut score	7.5	26.5
Sensitivity	.89	.82
Specificity	.71	.77
Child report		
Total sample		
*n* (positive; negative)	325; 324	323; 322
AUC	.71	.63
Optimal Cut score	6.5	32.50^a^
Sensitivity	.67	.61
Specificity	.64	.58
Boys		
*n* (positive; negative)	162; 145	161; 144
AUC	.74	.68
Optimal cut score	5.5.	24.5
Sensitivity	.73	.71
Specificity	.70	.61
Girls		
*n* (positive; negative)	163; 179	162; 178
AUC	.70	.62
Optimal cut score	7.5	36.5*
Sensitivity	.64	.61
Specificity	.63	.55
Teacher report		
Total sample		
*n* (positive; negative)	230; 340	227; 340
AUC	.76	.75
Optimal cut score	4.5	8.5
Sensitivity	.70	.71
Specificity	.73	.68
Boys		
*n* (positive; negative)	107; 156	108; 156
AUC	.76	.75
Optimal cut score	3.5	7.5
Sensitivity	.74	.71
Specificity	.64	.71
Girls		
*n* (positive; negative)	123; 184	119; 184
AUC	.77	.76
Optimal cut score	4.5	8.5
Sensitivity	.73	.74
Specificity	.69	.64
*Note.* SCAS = Spence Children’s Anxiety Scale; AUC = area under the curve; Brief SCAS = SCAS-P-8/SCAS-C-8/SCAS-T-8; SCAS (full) = SCAS-P/SCAS-C/SCAS-T-20.		
^a^ Not possible to achieve .60/.60 sensitivity/specificity balance.		

**Table 7 tbl7:** Logistic Regressions Examining the Contribution of Each Reporter Using the Brief Spence Children’s Anxiety Scale (SCAS)

Variable	*b* (Wald statistic)	Odds ratio (95% confidence interval)	*R*^2^	Model
Parent + teacher model				
Constant	−4.23 (153.39**)			
SCAS-P-8 (total score)	.34 (103.44**)	1.40 [1.31, 1.49]	.40 (Cox&Snell)	χ^2^(2) = 278.07**
SCAS-T-8 (total score)	.17 (24.63**)	1.18 [1.11, 1.26]	.54 (Nagelkerk)	
Parent + child model				
Constant	−3.19 (135.60**)			
SCAS-P-8 (total score)	.34 (128.97**)	1.40 [1.32, 1.48]	.35 (Cox&Snell)	χ^2^(2) = 273.23**
SCAS-C-8 (total score)	.03 (2.20, *p* = .14)	1.03 [.99, 1.08]	.47 (Nagelkerk)	
Teacher + child model				
Constant	−2.44 (106.49**)			
SCAS-T-8 (total score)	.25 (69.72**)	1.28 [1.21, 1.35]	.24 (Cox&Snell)	χ^2^(2) = 148.63**
SCAS-C-8 (total score)	.11 (25.75**)	1.12 [1.07, 1.16]	.33 (Nagelkerk)	
Parent + child + teacher model				
Constant	−4.24 (136.26**)			
SCAS-P-8 (total score)	.33 (85.76**)	1.39 [1.29, 1.48]		
SCAS-C-8 (total score)	.02 (.71, *p* = .40)	1.02 [.97, 1.08]	.40 (Cox&Snell)	χ^2^(3) = 261.40**
SCAS-T-8 (total score)	.16 (21.28**)	1.17 [1.09, 1.25]	.54 (Nagelkerk)	
** *p* < .001.				

## Footnotes

1 Differences on SCAS-P/C/T total scores among those classed as “higher/professional” compared with other socioeconomic groups, and among 7- to 8-year-olds compared with 9- to 11-year-olds were examined. No significant differences on SCAS total scores were found among those classed as “higher/professional” compared with other socioeconomic groups. No significant age effects were found on SCAS-P/T total scores, although significant age effects were found on the SCAS-C within the community sample, with total scores significantly higher among 7- to 8-year-olds than 9- to 11-year-olds, *t*[301] = 3.59, *p*<.001, *d* = .42, with the highest mean scores among 8-year-olds. Given the narrow age range of participants included in this study, however, subsequent analyses were not conducted separately for different age groups. With the aim of developing a brief measure, together with optimal cut-off scores for use in primary care and school settings, it would be impractical to provide gender differentiated cut-off scores for very narrow age bands.
