# Supplementary material for: Identifying Children With Anxiety Disorders Using Brief Versions of the Spence Children’s Anxiety Scale for Children, Parents, and Teachers
Source: Psychol Assess. 2018 Jun 14;30(10):1342–55. doi: 10.1037/pas0000570 (PMC6179143; doi:10.1037/pas0000570)
Supplement: Supplementary file 1 [file PAS-2017-1722IntSupp.docx]

**Supplemental Materials**

**Identifying Children With Anxiety Disorders Using Brief Versions of the**

**Spence Children’s Anxiety Scale for Children, Parents, and Teachers**

**by T. Reardon et al., 2018, *Psychological Assessment***

**http://dx.doi.org/10.1037/pas0000570**

Online Supplement 1

Internal consistency (Cronbach’s α) for brief and full versions of the SCAS in the two samples

|  | Community sample | Clinic-referred sample |
| --- | --- | --- |
| SCAS-P-8  SCAS-P | .82  .91 | .73  .89 |
| SCAS-C-8  SCAS-C | .84  .95 | .77  .91 |
| SCAS-T-8  SCAS-T-20 | .80  .89 | .85  .92 |

Online Supplement 2

Agreement between reporters (Pearson’s *r*) on the brief and full versions of the SCAS in the two samples

|  |  |  |
| --- | --- | --- |
|  | Community sample | Clinic-referred sample |
| Parent-Child |  |  |
| SCAS-P 8-  SCAS-C-8 | .40** | .34** |
|  |  |  |
| SCAS-P-  SCAS-C | .42** | .34** |
|  |  |  |
| Parent-Teacher |  |  |
| SCAS-P-8-  SCAS-T-8 | .32** | .28** |
|  |  |  |
| SCAS-P-  SCAS-T-20 | .29** | .21* |
|  |  |  |
| Teacher-Child |  |  |
| SCAS-T-8  SCAS-C-8 | .25** | .05, *p* = .46 |
|  |  |  |
| SCAS-T-20-  SCAS-C | .21** | .06, *p* =.39 |

***p*<.001

**p*<.01

Online Supplement 3

Convergent and Divergent validity indices for the brief and full SCAS

|  |  | Community sample | Clinic-referred sample |
| --- | --- | --- | --- |
|  | Parent report |  |  |
| Convergent validity | SCAS-P-8– SDQ-P-emotion | .76** | .62** |
|  | SCAS-P-8 SDQ-P-internalising | .70** | .58** |
|  | SCAS-P-SDQ-P-emotion | .76** | .59** |
|  | SCAS-P -SDQ-P internalising | .70** | .53** |
| Divergent validity | SCAS-P-8 -SDQ-conduct | .32** | .14* |
|  | SCAS-P-8 -SDQ-P-externalising | .34** | .10, *p* = .07 |
|  | SCAS-P - SDQ-P-conduct | 33** | .20** |
|  | SCAS-P - SDQ-P-externalising | 34** | .15* |
|  | Child report |  |  |
| Convergent Validity | SCAS-C-8– SDQ-C-emotion | .73** | .65** |
|  | SCAS-C-8- SDQ-C-internalising | .68** | .62** |
|  | SCAS-C- SDQ-C-emotion | .81** | .72** |
|  | SCAS-C- SDQ-C-internalising | .75** | .69** |
| Divergent validity | SCAS-C-8- SDQ-C-conduct | .24** | .27** |
|  | SCAS-C-8-SDQ-C-externalising | .33** | .31** |
|  | SCAS-C-SDQ-C-conduct | .31** | .29** |
|  | SCAS-C - SDQ-C-externalising | .40** | .34** |
|  | Teacher report |  |  |
| Convergent Validity | SCAS-T-8–SDQ-emotion | .74** | .73** |
|  | SCAS-T-8- SDQ-T-internalising | .64** | .65** |
|  | SCAS-T-20– SDQ-T-emotion | .76** | .75** |
|  | SCAS-T-20- SDQ-T-internalising | .65** | .68** |
| Divergent validity | SCAS-T-8-SDQ-T-conduct | .26** | .08, *p* = .22 |
|  | SCAS-T-8 -SDQ-T-externalising | .21** | .11, *p* = .10 |
|  | SCAS-T-20-SDQ-T conduct | .26** | .09, *p* = .18 |
|  | SCAS-T-20-SDQ-T externalising | .22** | .12, *p* = .08 |

SDQ-P/C/T-emotion = SDQ-P/T/C- emotional problems scale; SDQ-P/C/T-internalising = SDQ-P/T/C-internalising problems scale; SDQ-P/C/T-conduct = SDQ-P/T/C-conduct problems scale; SDQ-P/T/C-externalising = SDQ-P/C/T-externalising problems scale

***p*<.001

**p*<.01

Online Supplement 4

Sensitivity and specificity when using multiple informant versions of the brief SCAS

|  | Sensitivity | Specificity |
| --- | --- | --- |
| SCAS-P-8 + SCAS-T-8  (parent+teacher) | .93 | .54 |
| SCAS-P-8+ SCAS-C-8  (parent+child) | .95 | .54 |
| SCAS-P-T+ SCAS-C-  (teacher+child) | .89 | .48 |
| SCAS-P-8+SCAS-T-8+SCAS-C-8 (parent+teacher+child) | .97 | .42 |

*Note.*

Sensitivity/specificity values calculated using optimal cut-off scores for boys/girls identified in Table 6: SCAS-P-8, 7.5 (boys & girls); SCAS-C-8, 5.5 (boys), 7.5 (girls); SCAS-T-8, 3.5 (boys), 4.5 (girls).

*n* = 518 (i.e. participants with SCAS-P-8, SCAS-T-8, and SCAS-C-8 data available)

Online Supplement 5

Gender differences on the brief and full length SCAS

|  | Community sample | | | Clinic-referred sample | | |
| --- | --- | --- | --- | --- | --- | --- |
|  | Boys  Mean (SD) | Girls  Mean (SD) | *t* test (Cohen’s *d*) | Boys  Mean (SD) | Girls  Mean (SD) | *t* test (Cohen’s *d*) |
| SCAS-P-8  SCAS-P | 5.33 (3.35)  16.23 (10.85) | 6.03 (3.91)  20.13 (13.99) | *t*(356) = 1.79, *p* = .07  *t*(355) = 2.91** (*d* = 0.31) | 11.36 (4.58)  37.93 (16.64) | 12.33(4.43)  40.90 (15.90) | *t*(311) = 1.91, *p* = .06  *t*(310) = 1.61, *p* = .11 |
| SCAS-C-8  SCAS-C | 4.90 (4.47)  26.12 (20.43) | 6.84 (4.71)  36.18 (20.45) | *t*(322) = 3.78** (*d* = 0.42)  *t*(320) = 4.39** (*d* = 0.49) | 8.44 (4.70)  35.93 (17.85) | 9.93 (4.53)  43.57 (18.33) | *t*(323) = 2.90* (*d* = 0.32)  *t*(321) = 3.79** (*d* = 0.42) |
|  |  |  |  |  |  |  |
| SCAS-T-8 | 3.19 (2.98) | 3.56 (2.86) | *t*(338) = 1.18, *p* = .24 | 6.67 (4.38) | 7.92 (4.85) | *t*(228) = 2.03, *p* = .04 |
| SCAS-T-20 | 6.19 (6.20) | 7.39 (6.11) | *t*(338) = 1.80 *p* = .07 | 13.12 (9.53) | 15.96 (10.34) | *t*(225) = 2.14, *p* = .03 |

***p*<.001

**p*<.01
